# Minimally invasive management of vital teeth requiring root canal therapy

**DOI:** 10.1038/s41598-023-47682-9

**Published:** 2023-11-21

**Authors:** E. Karatas, M. Hadis, W. M. Palin, M. R. Milward, S. A. Kuehne, J. Camilleri

**Affiliations:** 1 Ataturk University, Erzurum, Turkey; 2 School of Dentistry, Institute of Clinical Sciences, College of Medical and Dental Sciences, University of Birmingham, 5, Mill Pool Way Edgbaston, Birmingham, B5 7EG UK; 3 School of Science and Technology, Nottingham Trent University, Nottingham, UK

**Keywords:** Materials science, Biomaterials, Biomedical materials, Dentistry, Dental materials, Endodontics, Microbiology, Bacteria

## Abstract

The present study aimed to investigate the possible use of a non-instrumentation technique including blue light irradiation for root canal cleaning. Extracted human single rooted teeth were selected. Nine different groups included distilled water, NaOCl, intra-canal heated NaOCl, and NaOCl + EDTA irrigation after either instrumentation or non-instrumentation, and a laser application group following non-instrumentation technique. The chemical assessment of the root canal dentine was evaluated using energy dispersive spectroscopy (EDS) and Fourier transform infrared (FT-IR) spectroscopy. Surface microstructural analyses were performed by using scanning electron microscopy (SEM). The antimicrobial efficacy of different preparation techniques was evaluated using microbial tests. Light application didn’t change the calcium/phosphorus, carbonate/phosphate and amide I/phosphate ratios of the root canal dentin. The root canal dentin preserved its original chemistry and microstructure after light application. The instrumentation decreased the carbonate/phosphate and amide I/phosphate ratios of the root canal dentin regardless of the irrigation solution or technique (*p* < 0.05). The application of light could not provide antibacterial efficacy to match the NaOCl irrigation. The NaOCl irrigation both in the non-instrumentation and instrumentation groups significantly reduced the number of bacteria (*p* < 0.05). The use of minimally invasive root canal preparation techniques where the root canal is not instrumented and is disinfected by light followed by obturation with a hydraulic cement sealer reduced the microbial load and preserved the dentin thus may be an attractive treatment option for management of vital teeth needing root canal therapy.

## Introduction

Root canal shaping and irrigation procedures are considered the most important steps in endodontic treatment^1^. The main aim of these procedures is the removal of necrotic tissue, microorganisms, and infected dentin from the root canal system^1^. For this reason, numerous root canal instrumentation and irrigation techniques, instruments, and devices have been proposed to effectively eliminate intra-canal debris and pathogens and manage apical periodontitis^1–8^.

Root canal instrumentation alone is unable to clean the root canal system adequately especially in the apical canal region^9^. Thus, root canal irrigation is a mandatory step during root canal treatment, as it enables the cleaning of parts of the root canal which are left untouched after mechanical instrumentation. Root canal instrumentation may result in procedural errors such as ledging, apical transportation, instrument fracture, elbow formation, and strip perforation^10^. Some complications such as extrusion of the irrigant or necrotic infected debris into the periapical area, air emphysema, splash of the irrigant into the operator’s or patient’s eye, and allergic reactions to the irrigant may occur during the irrigation of the root canals^11^. Moreover, instrumentation and irrigation procedures alter the surface morphology and the chemical and mechanical properties of the root canal dentin^12–14^.

Instrumentation of the root canals results in the formation of a smear layer that contains pulp tissue remnants, bacteria, infected dentin, and dead bacterial cells. It is well known that bacteria may survive and proliferate into the dentinal tubules in the presence of a smear layer^15^. Additionally, it may limit the optimum penetration of antibacterial solutions and can compromise adequate sealing by acting as a barrier between the root canal wall and filling materials^16^. It is possible to remove the smear layer from the root canal walls by using irrigation solutions that are primarily calcium chelators. However, irrigation procedures have some deleterious effects on root canal walls such as a decrease in the modulus of elasticity, microhardness, flexural strength, inorganic content, and the organic–inorganic ratio of the dentin^17^. These changes may jeopardise the success of root canal treatment via decreasing the fracture strength of root canal treated teeth. Therefore, the investigation of innovative root canal cleaning techniques, materials, and devices continues.

Cleaning of the root canal system without instrumentation and the creation of a smear layer may be possible with non-instrumentation techniques. Several studies evaluated different non-instrumentation techniques and reported promising results^18–20^. However, all these studies used NaOCl irrigation in the non-instrumentation groups for the disinfection of the root canals, which, has numerous deleterious effects on root canal dentin^17^. Therefore, a minimally invasive root canal cleaning technique that does not include instrumentation and chemical irrigation of the root canals is necessary.

This study proposes an alternative antimicrobial approach to root canal decontamination using the direct application of blue light in the spectrum of 400–470 nm which has been reported to have antibacterial effects against a variety of pathogens^8^. Previous studies.^8^ evaluated the antimicrobial efficacy of blue light and demonstrated that its application significantly eliminated endodontic pathogens such as *Enterococcus faecalis*, methicillin-resistant *Streptococcus aureus*, and *Prevotella intermedia*. The blue light exerts its antibacterial effect by excitation of endogenous microbial chromophores such as flavins and porphyrins which subsequently results in the generation of reactive oxygen species (ROS)^7^. Several studies demonstrated that direct light application is useful for the disinfection of dental tissues and can inhibit a wide range of bacterial species residing within biofilms^7,8,21–23^. Microorganisms also do not become resistant to blue light in the long term ^24^. However, the antimicrobial effects of blue light depend on several factors, namely the irradiance of the light and the exposure duration which determine the energy delivered to the target site. The delivery of light for intra-canal irradiation is complicated by the complex architecture and microstructure of dental tissues which will absorb and scatter light to limit the amount of light that can be delivered to microorganisms for effective antimicrobial effects. Currently, it is not known if the direct application of light can obtain adequate bacterial reduction after intra-canal irradiation. Additionally, the effect of direct light application on the chemical and mechanical properties of dental tissues including the root canal dentin has not been well established. The present study aimed to investigate the possible use of a non-instrumentation technique including blue light irradiation for root canal cleaning without instrumentation and chemical irrigation followed by obturation using a hydraulic cement sealer for the management of vital teeth requiring root canal therapy. Thus, the deleterious effects of instrumentation and irrigation procedures on root canal dentin would be excluded. This methodology was compared to standard root canal instrumentation and irrigation protocols and obturation with gutta-percha and sealer.

## Results

### Light transmission and irradiance measurement

**Figure 1:**
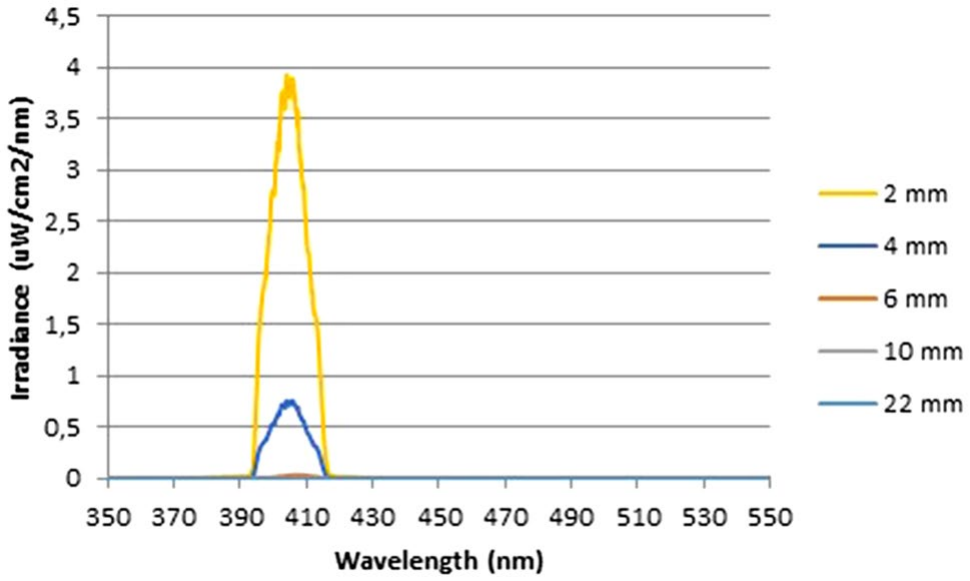
Light transmission levels (µW/cm^2^/nm) for (**a**) 2 and 4 mm samples and (**b**) 6, 10 and 22 mm samples. The absolute irradiance was calculated using the area under the curve.

Light transmission measurements revealed that the irradiances delivered to the lower surfaces of 2, 4, 6, 10, and 22-mm samples for the highest power settings were 3.99, 0.55, 0.04, 0.01, and 0.002 mW/cm^2^ respectively (Fig. 1). The light transmission was negligible in samples with a thickness of more than 4 mm. Therefore, it has been decided that both intra-canal and extra-canal applications of light should be considered to able to irradiate whole the root canal. For this reason, laser fibre irradiance measurements were performed. According to the results, in the highest current setting of the laser device, the irradiance was 38,072 and 1032 mW/cm^2^ for 105 and 200 µm fibres, respectively.

### Analysis of the heat changes

**Figure 2:**
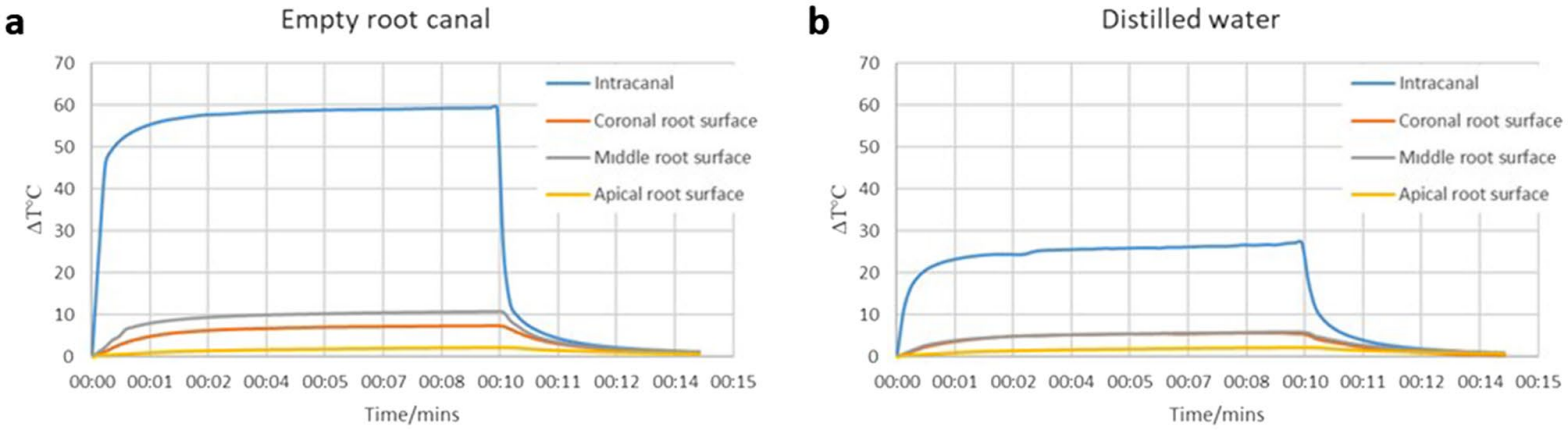
The change in temperature recorded during the intracanal laser application with 200 µm fibre either in an empty dry root canal (**a**) or with the presence of 0.1 mL of distilled water (**b**).

The intra-canal and external root surface heat changes during the laser application with 200 µm are shown in Fig. 2. The temperature inside the root canal increased immediately with the application of the laser and reached a maximum rise of 59 °C when the root canal was empty. However, the maximum temperature rise recorded during the laser application with the presence of distilled water inside the root canal was only 27 °C. Similarly, while the highest increase in temperature on the external root surface was 10.8 °C in the empty root canal, it was only 5.9 °C in the sample the root canal was filled with distilled water. The highest temperature change on the external root surface was recorded at the middle root level as the tip of the laser fibre was at 5 mm level from the apex.

Since the increase in temperature in the empty root canal sample was higher than the biological limits for outer surface of the root canal (10 °C), it has been decided to apply the laser (200 µm fibre) inside the root canal with the presence of distilled water throughout the study.

**Figure 3:**
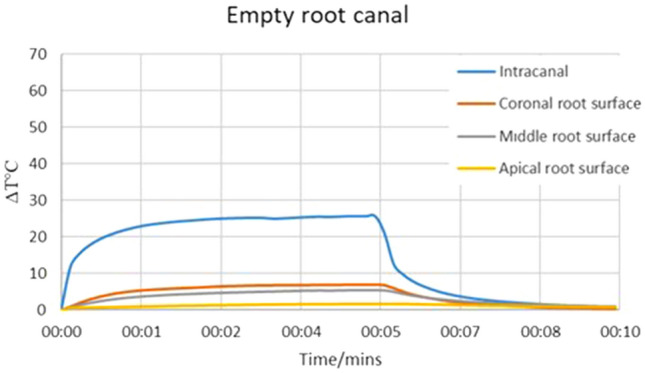
The change in temperature recorded during the extra-canal laser application (from the root canal orifice) with 105 µm fibre in an empty dry root canal.

The temperature rise in the laser application from the root canal orifice can be seen in Fig. 3. The maximum increase in temperature was 25 °C and 6.8 °C for intra-canal and external root surface measurements respectively. The highest change in temperature on the external root surface (6.8 °C) was observed at the coronal level. Since the maximum temperature changes were within the biological limits, it has been decided that filling the root canal with distilled water is not necessary and it is safe to apply the laser (105 µm fibre) from the root canal orifice while the root canal was dry and empty.

**Figure 4:**
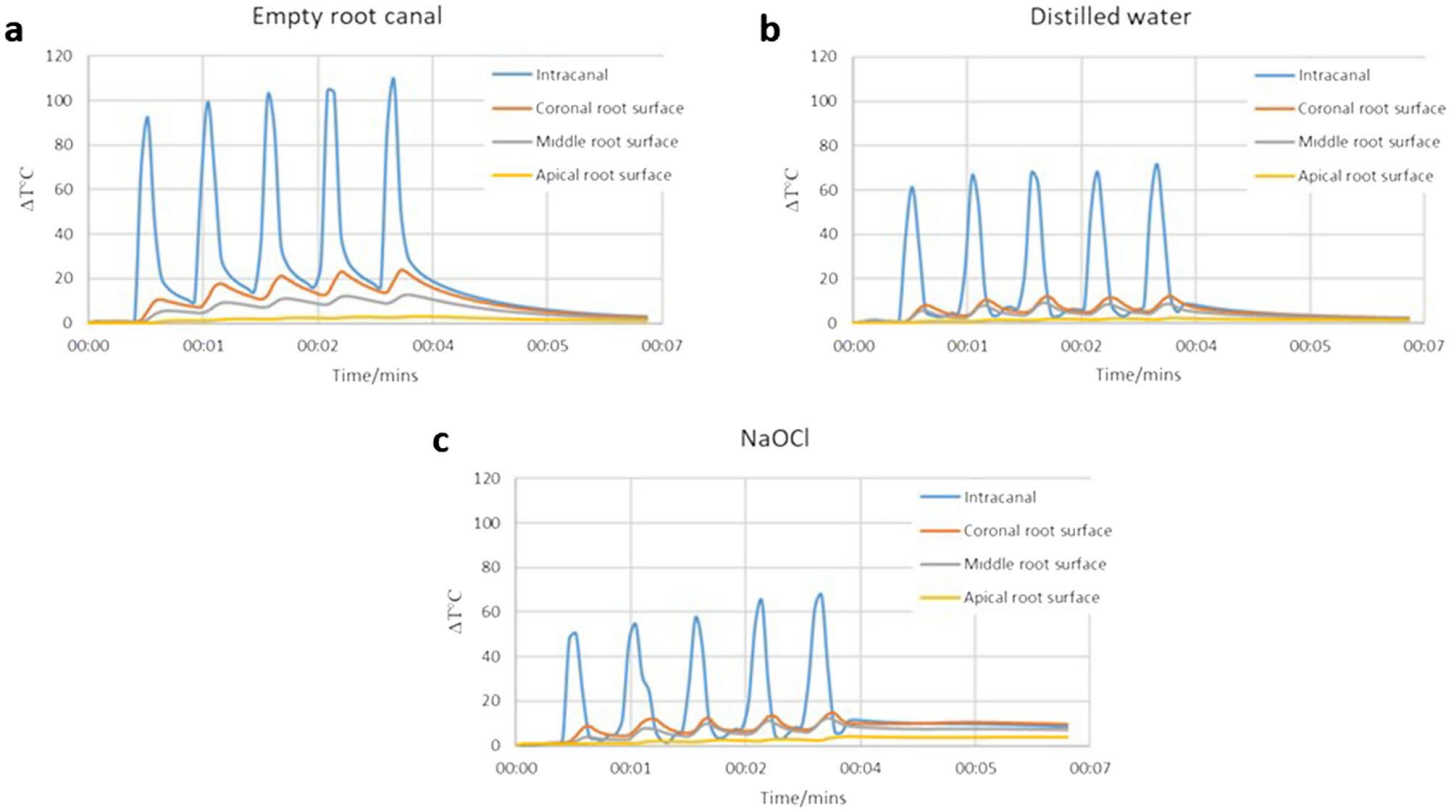
The changes in temperature recorded during intra-canal heat application with SuperEndo either in an empty dry root canal (**a**), with the presence of distilled water (**b**), or NaOCl (**c**).

The temperature changes during the intra-canal application of heat with SuperEndo can be seen in Fig. 4. In all the samples, the temperature both inside the root canal and external root surface increased immediately with the activation of the heat source, and an immediate decrease was also observed once the heat source was deactivated. However, the maximum rise in the temperature inside the root canal was 109 °C and 23.7 °C for intra-canal and external root surface measurements in the empty root canal sample. In contrast, the maximum intra-canal temperature rises were 71 °C and 67 °C for the distilled water and NaOCl samples respectively. External root surface temperature change measurements were also relatively similar for the distilled water and NaOCl samples. The maximum temperature rise on the external root surface was 12.2 °C and 14.4 °C for the distilled water and NaOCl samples respectively. It has been demonstrated that the temperature rises were above the biological limits for all the 3 samples. Most importantly, the temperature of the root surface could not be restored for 2 min following the completion of the 5 cycles of the irrigation procedure, especially in distilled water and NaOCl samples.

### SEM evaluation

**Figure 5:**
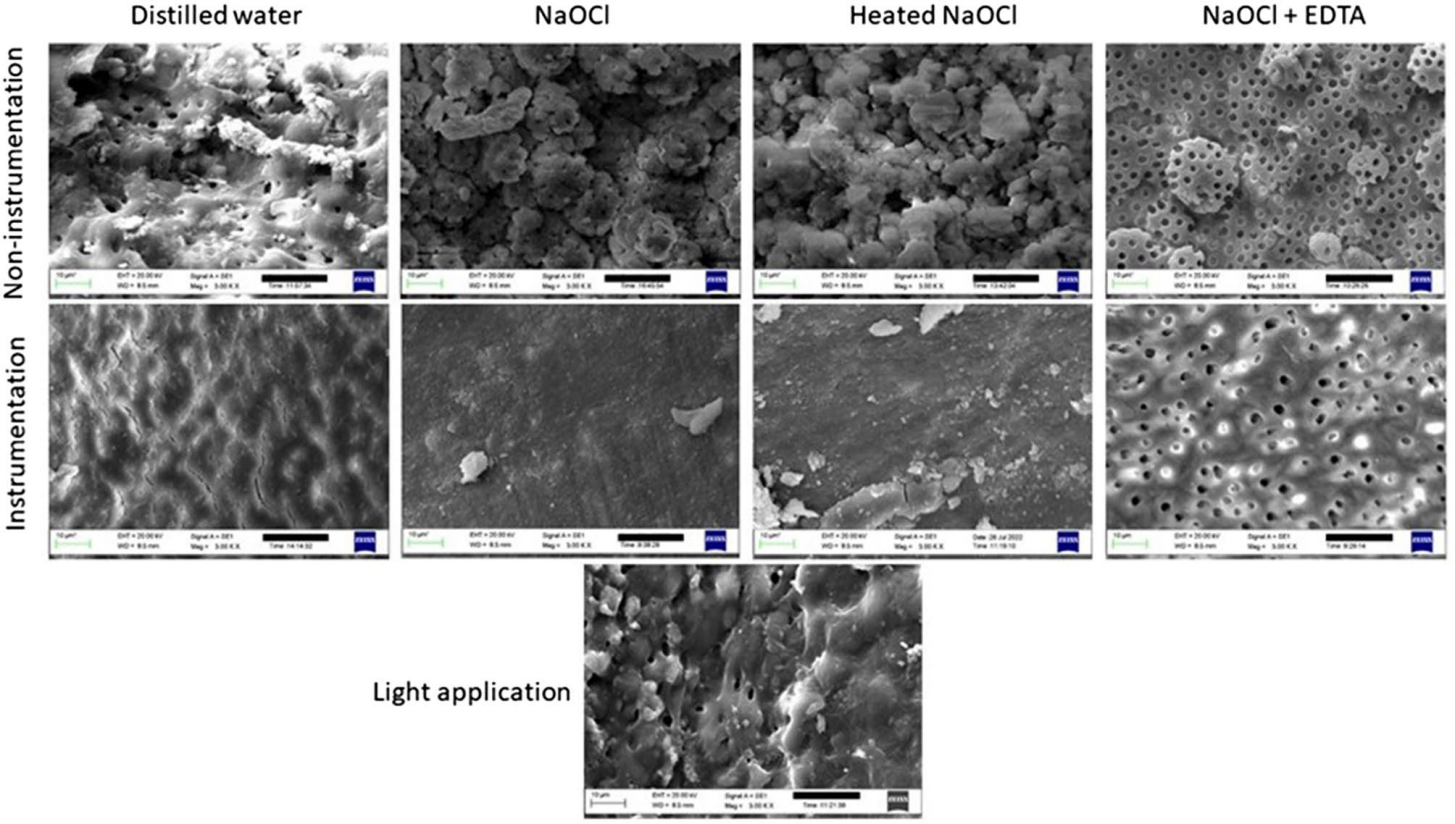
SEM images of root canal surface at ×3 K magnification after different preparation procedure.

Figure 5 shows the SEM images for the groups. The SEM images showed that root canal dentin preserved its original form in the light application group without any damage as in the non-instrumentation distilled water group. After root canal instrumentation, the dentin surface was covered with a smear layer which the distilled water and NaOCl irrigation were unable to remove. Final irrigation with EDTA removed the smear layer and presented clean walls with opened and widened dentinal tubules. Although instrumentation was not performed in the non-instrumentation groups, a smear layer was formed in the NaOCl and heated NaOCl groups. This erosive effect was more evident in the non-instrumentation heated NaOCl group, showing a tunnelling erosion pattern on the dentin, with enlarged, interconnected dentinal tubular spaces. Final irrigation with EDTA clearly showed the loss of integrity of the inter-tubular dentin.

### EDS analysis

Table 1 shows the median and min–max values for the groups. NaOCl irrigation of the root canals without a final flush of EDTA resulted in a decrease in the Ca/P ratio and the reduction was statistically significant when intra-canal heating was performed in the non-instrumentation group (*p* < 0.05). However, the difference was not statistically significant when the instrumentation was followed by NaOCl or intra-canally heated NaOCl (*p* > 0.05). A final flush of the root canals using EDTA recovered the Ca/P ratio as the difference was not statistically significant when comparing both the instrumentation + EDTA and non-instrumentation EDTA groups with the distilled water groups. The light application didn’t change the Ca/P ratio of the root canal wall as there were no statistically significant differences when compared with the other groups except the non-instrumentation + heated NaOCl group.

**Table 1:** Median and min–max Ca/P ratios according to the root canal preparation groups (*n* = 18).

	Median	Min–Max
Instrumentation + Distilled water	1.97^a^	1.7–2.7
Instrumentation + NaOCl	1.95^ab^	1.7–2.6
Instrumentation + NaOCl (heated)	1.90^ab^	1.8–2.1
Instrumentation + NaOCl + EDTA	2.03^a^	1.7–3.9
Non-instrumentation + Distilled water	2.25^a^	1.8–4.1
Non-instrumentation + NaOCl	2.05^a^	1.7–2.8
Non-instrumentation + NaOCl (heated)	1.78^b^	1.5–2
Non-instrumentation + NaOCl + EDTA	2.25^a^	1.7–3.5
Light	2.30^a^	1.4–4.5

Values with the same superscript letter are not statistically different at the 5% level.

### FT-IR analysis

Table 2 shows values for the ratios of carbonate/phosphate and amide I/phosphate. Laser application didn’t change the carbonate/phosphate and amide I/phosphate ratios of the root canal dentin because there was no statistically significant difference between the laser and non-instrumentation + DW groups (*p* > 0.05). Another important finding would be that the instrumentation decreased the carbonate/phosphate and amide I/phosphate ratios of the root canal dentin regardless of the irrigation solution or technique. Additionally, NaOCl irrigation, except the non-instrumentation + NaOCl + EDTA group, decreased the carbonate/phosphate and amide I/phosphate ratios significantly when compared with the distilled water irrigation group (*p* < 0.05). Interestingly, in the non-instrumentation groups, the final flush of the root canals using EDTA recovered the carbonate/phosphate and amide I/phosphate ratios.

**Table 2:** Carbonate/phosphate and amide 1/phosphate findings according to the FT-IR spectroscopic analysis (*n* = 18).

	Mean	SD
**Carbonate/phosphate ratio**		
Instrumentation + DW	0.42^b^	0.02
Instrumentation + NaOCl	0.35^b^	0.04
Instrumentation + NaOCl (heated)	0.36^b^	0.03
Instrumentation + NaOCl + EDTA	0.30^b^	0.06
Non-instrumentation + DW	1.22^a^	1.16
Non-instrumentation + NaOCl	0.34^b^	0.02
Non-instrumentation + NaOCl (heated)	0.37^b^	0.04
Non-instrumentation + NaOCl + EDTA	1.04^a^	0.40
Light	1.05^a^	0.58
**Amide 1/phosphate ratio**		
Instrumentation + DW	0.58^b^	0.45
Instrumentation + NaOCl	0.44^b^	0.05
Instrumentation + NaOCl (heated)	0.62^b^	0.15
Instrumentation + NaOCl + EDTA	0.50^b^	0.10
Non-instrumentation + DW	2.97^a^	2.88
Non-instrumentation + NaOCl	0.47^b^	0.03
Non-instrumentation + NaOCl (heated)	0.45^b^	0.04
Non-instrumentation + NaOCl + EDTA	2.08^a^	0.84
Light	2.12^a^	1.31

*DW: Distilled water, Values with the same superscript letter are not statistically different at the 5% level.*

### SEM evaluation for the obturation groups

**Figure 6:**
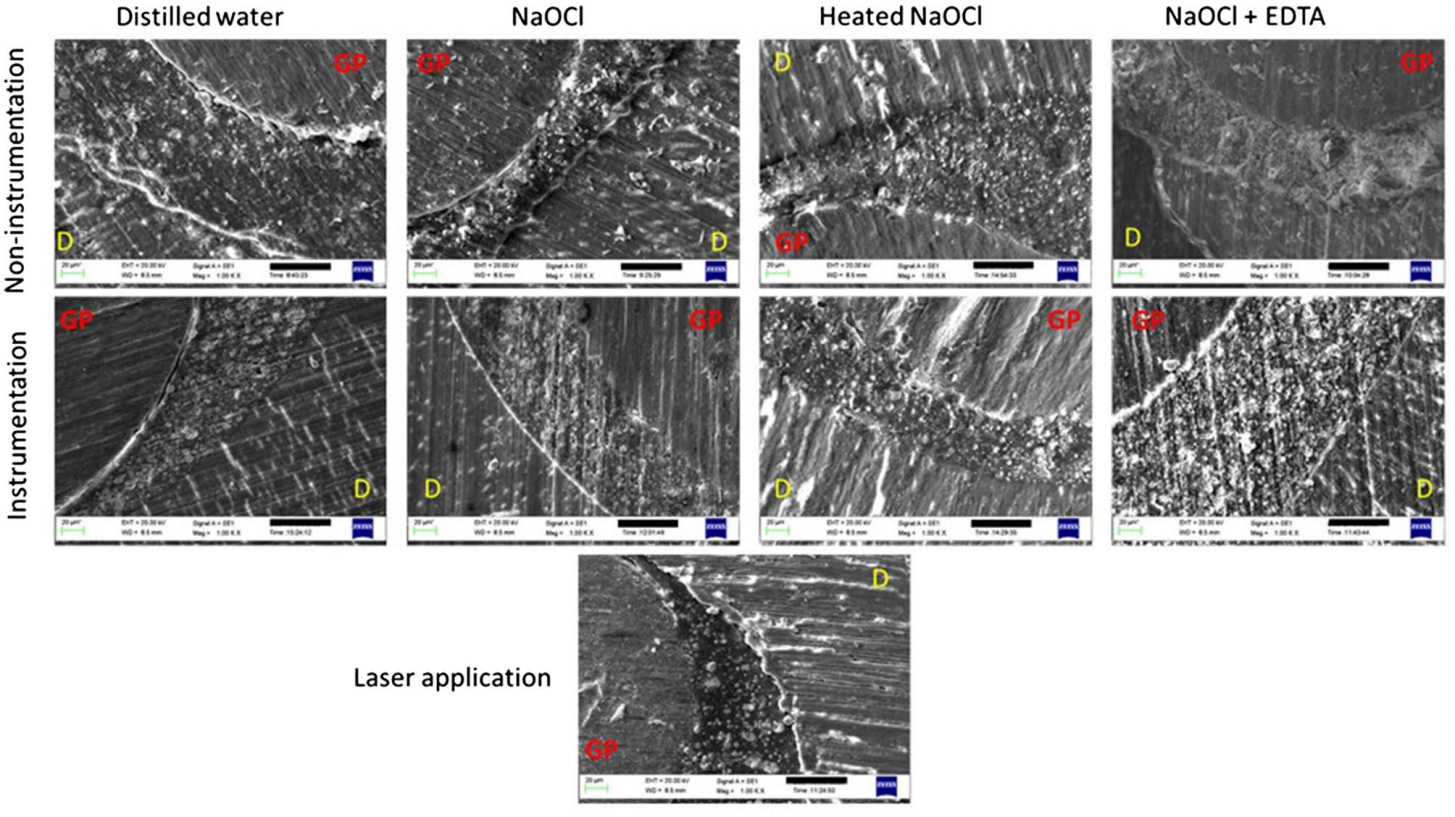
SEM images of horizontal root sections at ×1 K magnification after obturation with AH Plus following different preparation procedures. *GP: gutta-percha, D: the dentin*.

**Figure 7:**
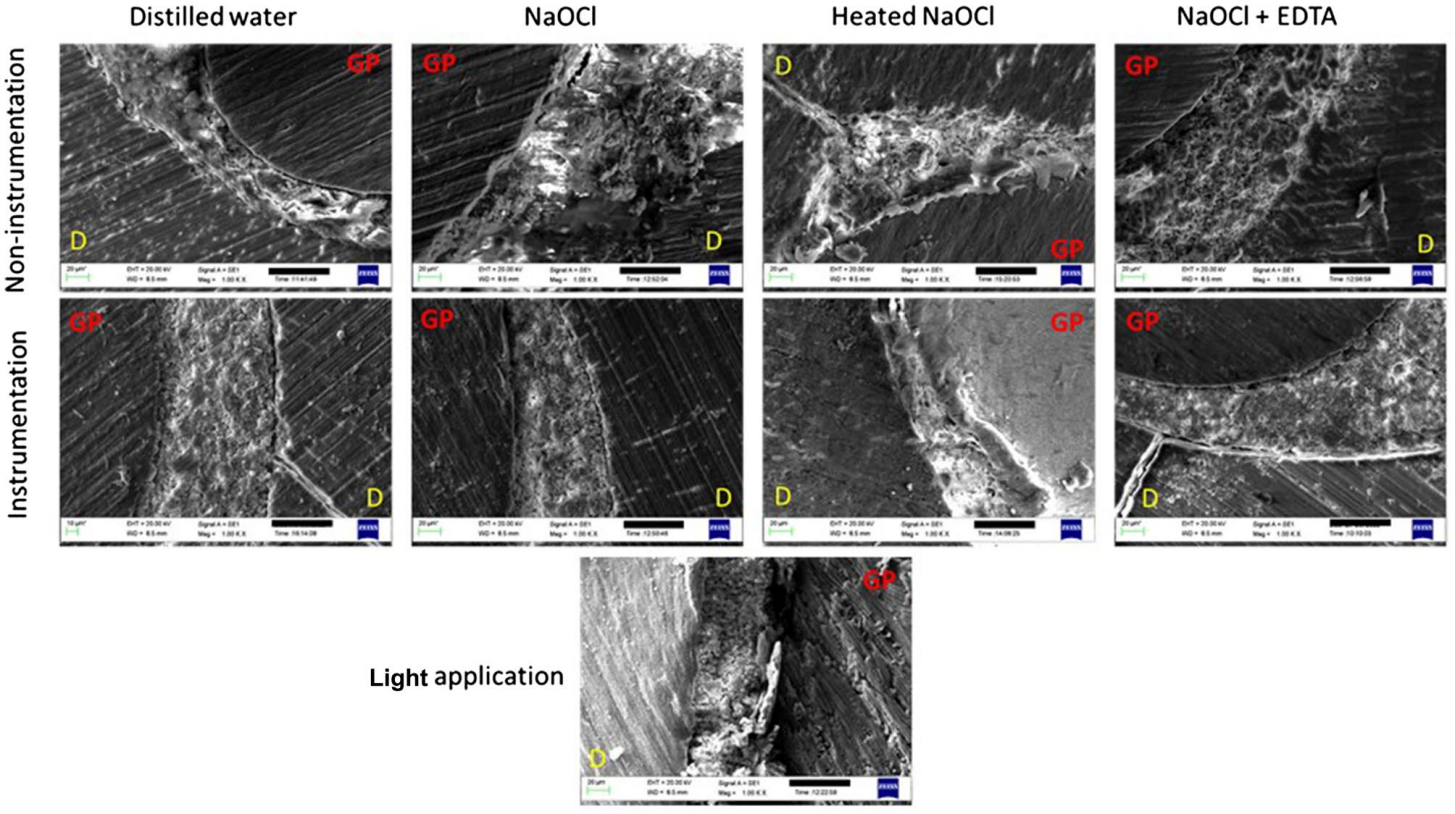
SEM images of horizontal root sections at ×1 K magnification after obturation with BioRoot RCS RCS following different preparation procedures*. GP: gutta-percha, D: dentin*.

Figures 6 and 7 show the dentin–sealer and sealer–gutta-percha interfaces for the sealers. According to the images, AH Plus exhibited adequate marginal adaptation with both the dentin and gutta-percha irrespective of the preparation protocol. However, the BioRoot RCS sealer did not show good marginal adaptation with root canal surface in any of the samples. There were slight interfacial gaps in almost all the BioRoot RCS sealer samples. Additionally, BioRoot RCS sealer revealed a relatively uniform distribution of irregular micro-sized particles aggregated in clusters. In contrast, the AH Plus was more compact and the surface of the sealer was more regular compared to the BioRoot RCS.

### EDS analysis for the obturation groups

The median Ca/P values according to the intra-group EDS analyses can be shown in Table 3. According to the findings, there was no statistically significant difference among the different preparation techniques neither in the AH Plus nor in the BioRoot RCS groups. However, when an overall inter-group comparison was made for the AH Plus and BioRoot RCS groups, it has been found that there was a statistically significant difference between the groups (*p* < 0.05). While the average Ca/P ratio for the AH Plus group was 1.899, it was 2.369 for the BioRoot RCS group (Table 4). BioRoot RCS had higher Ca/P ratios due to the presence of Ca ions within its composition and the penetration ability of the sealer into the dentinal tubules.

**Table 3:** Intra-group comparisons of Ca/P ratios according to the preparation groups either for AH Plus or BioRoot RCS samples (*n* = 18).

	Median Ca/P ratio	Min–Max Ca/P ratio
**AH Plus**		
Instrumentation + DW	1.90	1.8–2
Instrumentation + NaOCl	1.92	1.6–2.2
Instrumentation + NaOCl (heated)	1.94	1.9–2
Instrumentation + NaOCl + EDTA	1.88	1.8–2
Non-instrumentation + DW	1.94	1.8–2.1
Non-instrumentation + NaOCl	1.84	1.7–1.9
Non-instrumentation + NaOCl (heated)	1.89	1.9–2
Non-instrumentation + NaOCl + EDTA	1.90	1.9–2
Light	1.82	1.8–2
**BioRoot RCS**		
Instrumentation + DW	2.14	1.9–2.8
Instrumentation + NaOCl	2.31	2–2.8
Instrumentation + NaOCl (heated)	2.69	1.8–3.1
Instrumentation + NaOCl + EDTA	2.07	2–2.4
Non-instrumentation + DW	2.09	1.9–2.1
Non-instrumentation + NaOCl	2.33	1.8–2.9
Non-instrumentation + NaOCl (heated)	2.57	2.4–2.7
Non-instrumentation + NaOCl + EDTA	2.53	2–3.3
Light	2.51	1.8–3.9

*DW: Distilled water.*

**Table 4:** Inter-group comparison of the Ca/P ratios according to the type of the sealer.

	N	Median Ca/P ratio	Min–Max	*P* value
AH Plus	54	1.899	1.63–2.15	*0.000*
BioRoot RCS	54	2.369	1.8–3.9	

### FT-IR analysis for obturation groups

The median carbonate/phosphate and amide I/phosphate values for the AH Plus and BioRoot RCS groups according to the intra-group FT-IR analyses can be shown in Table 5. In the AH Plus group, the light application did not change the carbonate/phosphate ratio significantly. In contrast, the amide I/phosphate values for the light group were significantly higher than the instrumentation + NaOCl heated group (*p* < 0.05). Additionally, the instrumentation followed by heated NaOCl irrigation protocol significantly lowered both the carbonate/phosphate and amide I/phosphate values when compared with non-instrumentation DW group (*p* < 0.05).

**Table 5:** Intra-group comparisons of carbonate/phosphate and amide 1/phosphate findings according to the FT-IR analysis (*n* = 18).

	Median (Min–Max) carbonate/phosphate ratio	Median (Min–Max) amid/phosphate ratio
**AH Plus**		
Instrumentation + DW	0.45^ab^ (0.38–0.58)	0.64^abc^ (0.42–1)
Instrumentation + NaOCl	0.42^ab^ (0.38–0.51)	0.62^bc^ (0.58–0.68)
Instrumentation + NaOCl (heated)	0.39^b^ (0.36–0.58)	0.59^b^ (0.56–0.65)
Instrumentation + NaOCl + EDTA	0.48^a^ (0.44–0.53)	0.68^a^ (0.61–0.8)
Non-instrumentation + DW	0.48^a^ (0.37–0.67)	0.73^a^ (0.59–1)
Non-instrumentation + NaOCl	0.43^ab^ (0.39–0.5)	0.63^abc^ (0.60–0.71)
Non-instrumentation + NaOCl (heated)	0.43^ab^ (0.39–0.51)	0.65^abc^ (0.55–0.69)
Non-instrumentation + NaOCl + EDTA	0.52^a^ (0.39–0.59)	0.68^ac^ (0.58–0.76)
Light	0.46^ab^ (0.4–0.51)	0.67^ac^ (0.5–0.72)
**BioRoot RCS**		
Instrumentation + DW	0.38^ab^ (0.34–0.43)	0.70^ab^ (0.55–0.75)
Instrumentation + NaOCl	0.38^b^ (0.30–0.44)	0.66^ab^ (0.59–0.73)
Instrumentation + NaOCl (heated)	0.39^b^ (0.33–0.43)	0.58^b^ (0.52–0.89)
Instrumentation + NaOCl + EDTA	0.39^ab^ (0.35–0.58)	0.63^b^ (0.57–0.65)
Non-instrumentation + DW	0.42^a^ (0.39–0.48)	0.70^a^ (0.62–0.77)
Non-instrumentation + NaOCl	0.38^ab^ (0.31–0.59)	0.63^ab^ (0.51–0.75)
Non-instrumentation + NaOCl (heated)	0.43^ab^ (0.35–0.46)	0.60^b^ (0.55–0.67)
Non-instrumentation + NaOCl + EDTA	0.40^ab^ (0.18–0.45)	0.68^ab^ (0.61–0.73)
Light	0.39^ab^ (0.35–0.47)	0.67^ab^ (0.42–0.73)

Values with the same superscript letter are not statistically different at the 5% level.

In the BioRoot RCS groups, light application did not significantly change the carbonate/phosphate and amide I/phosphate ratios when compared with the non-instrumentation DW group (*p* > 0.05). However, carbonate/phosphate ratio was significantly decreased by both the NaOCl and NaOCl heated irrigation followed by the instrumentation protocol when compared with the non-instrumentation DW group (*p* < 0.05). The amide I/phosphate ratio was decreased significantly following NaOCl heated irrigation protocol irrespective of the instrumentation protocol (*p* < 0.05).

Interestingly, instrumentation followed by heated NaOCl irrigation significantly lowered the amide I/phosphate ratio both in the AH Plus and BioRoot RCS groups, when compared with the non-instrumentation DW group which can be considered as the control group (*p* < 0.05).

According to the inter-group comparison, the carbonate/phosphate ratio for the AH Plus group was significantly higher than the BioRoot RCS group (*p* < 0.05) (Table 6). However, there was no statistically significant difference between both groups in terms of the amide I/phosphate ratio (*p* > 0.05).

**Table 6:** Inter-group comparison of carbonate/phosphate and amide 1/phosphate findings according to the FT-IR analysis.

	N	Median (Min–Max) carbonate/phosphate ratio	Median (Min–Max) amide I/phosphate ratio
AH Plus	162	0.45 (0.36–0.67)	0.65 (0.42–1)
BioRoot RCS	162	0.40 (0.18–0.59)	0.64 (0.42–0.89)
*P* value		*0.000*	*0.174*

### Agar diffusion assays

No zones of inhibition were found with 2, 3 and 5 min light applications using the 200 µm fibre or with 2 and 3 min applications of the 105 µm fibre. It was demonstrated that an inhibition zone was obtained with the application of light for at least 10 min with the 200 µm fibre and 5 min with 105 µm fibre. The diameters of the zones were 1.5 mm (688 pixels) and 3.6 mm (1613 pixels) for the 200 µm and 105 µm fibres, respectively.

### Antibacterial effect of different root canal preparation procedures

Tables 7 and 8 show the median and min–max bacterial counts of *E. faecalis* and *S. mutans* species according to the groups. The CFU counts in the negative control group were 10^5^ CFUs/mL for both *E. faecalis* and *S. mutans* demonstrating that there was a decrease of 3 logs during the incubation and inoculation processes, which is considered to be sufficient for eradication^25^.

**Table 7:** CFU counts of *E. faecalis* according to the groups (*n* = 9).

Groups	Median	Min–Max
Negative control group	1.06 × 10^5a^	5.5 × 10^4^–1.6 × 10^5^
Instrumentation + Distilled water	1 × 10^4ab^	4.6 × 10^3^–1.6 × 10^4^
Instrumentation + NaOCl	0^b^	0
Instrumentation + NaOCl (heated)	0^b^	0
Instrumentation + NaOCl + EDTA	0^b^	0
Non-instrumentation + Distilled water	6.8 × 10^4a^	2.2 × 10^4^–1.2 × 10^5^
Non-instrumentation + NaOCl	0^b^	0
Non-instrumentation + NaOCl (heated)	0^b^	0
Non-instrumentation + NaOCl + EDTA	5.5^b^	0–33
Light+ Distilled water	4.3 × 10^4a^	3 × 10^3^–5.8 × 10^3^

Values with the same superscript letter are not statistically different at the 5% level.

**Table 8:** CFU counts of *S. mutans* according to the groups (*n* = 9).

Groups	Median	Min–Max
Negative control group	1.02 × 10^5a^	8 × 10^4^–1.3 × 10^5^
Instrumentation + Distilled water	1.1 × 10^2ab^	6.6 × 10^1^–1.6 × 10^2^
Instrumentation + NaOCl	0^b^	0
Instrumentation + NaOCl (heated)	0^b^	0
Instrumentation + NaOCl + EDTA	0^b^	0
Non-instrumentation + Distilled water	4.9 × 10^2a^	3 × 10^2^–8.1 × 10^2^
Non-instrumentation + NaOCl	3.6^b^	0–33
Non-instrumentation + NaOCl (heated)	1.9^b^	0–17
Non-instrumentation + NaOCl + EDTA	0^b^	0
Light + Distilled water	4.8 × 10^2a^	3.6 × 10^2^–6.3 × 10^2^

Kruskal–Wallis *h* test was performed. Values with the same superscript letters are not significantly different at *P* = 0.05.

According to the results, distilled water irrigation in either the non-instrumentation or in the instrumentation groups failed to reduce the bacterial load significantly for both *E. faecalis* and *S. mutans* (*P* < 0.001). This finding was the same for the light group as the number of CFUs in this group was not significantly different from the negative control group (*P* > 0.05). However, NaOCl irrigation both in the non-instrumentation and instrumentation groups significantly reduced the number of CFUs proving the strong antibacterial efficacy of NaOCl in this experimental model. Interestingly, there was no statistically significant difference between the instrumentation + distilled water and NaOCl groups in terms of the number of CFUs, demonstrating that instrumentation is also an important factor in the removal of bacteria from the root canal system and can provide similar results to NaOCl irrigation.

## Discussion

In the present study, different instrumentation and irrigation protocols were tested to evaluate their effects on root canal dentin chemistry and microstructure and to compare their antimicrobial efficacy. Additionally, a novel non-instrumentation protocol for root canal disinfection using blue light irradiation was investigated. For this reason, SEM, EDS and FT-IR evaluations were performed to analyse root canal dentin following different root canal preparations techniques. Furthermore, the antimicrobial efficacy of the different root canal preparation techniques was evaluated by microbiological experiments.

Previously, it has been shown that light can be transmitted through the dentin which is in accordance with the findings of the present study^23^. However, the present data demonstrated that light cannot be transmitted through the dentin in root samples more than 4 mm long. Therefore, for irradiation in deeper areas of the root canal, it is necessary to implement a multi-irradiation protocol to ensure sufficient energy and photon delivery for antimicrobial effects. For this reason, both intra-canal and extra-canal applications of light were considered able to transmit light to the apical parts of the root canal and irradiate the entire root canal space. Previous studies reported that the bacterial penetration depth into the dentinal tubules is within a range between 184 and 1643 µm^6,26,27^. Therefore, 4 mm light transmission through the dentin was sufficient for disinfection of the deeper parts of the root canal dentin. However, light application caused an increase in the temperature of the external root surface (10.8 °C) which was higher than the biological limits (+ 10 °C). It is believed that a 10 °C increase is a critical level and a heat increase above this level may result in irreversible periodontal injuries^28,29^. Thus, it has been decided to apply the light inside the root canal with the presence of distilled water which lowered the heat increase within the biological limits (5.9 °C).

The light application did not damage the root canal dentin as indicated by the scanning electron microscopy showing unchanged microstructure. There was no pulpal debris or smear layer formation and the root canal dentin preserved its original form showing intact calcospherites and smooth surface morphology. This finding is in accordance with previous studies that reported that the unprepared dentin surface was composed of exposed calcospherites with preserved predentin^30,31^. In contrast, instrumentation or NaOCl irrigation protocols damaged the root canal dentin and changed its original morphology. Especially, NaOCl irrigation caused a smear layer formation irrespective of the instrumentation protocol and showed an irreversible destructive effect on mineralized dentin. This finding was expected as the NaOCl has a proven organic matter dissolving ability and was capable of removing the organic phase from the superficial subsurface of mineralized dentin which resulted in the deterioration of predentin^32^. Removal of the organic phase from the mineralized dentin can make it more brittle^33^ as the collagen matrix provides fatigue resistance, toughness, and viscoelasticity to the dentin^34,35^. Moreover, the dissolving of the encapsulated collagen molecules by NaOCl results in unbound apatite crystallites remaining within the collagen-depleted “ghost mineralized dentin matrix”^36^ which may enhance the permeability of dentin^37^. It is well known that the mineralized dentin is protected from thermal denaturation and enzymatic degradation by apatite crystallites within the interfibrillar and intrafibrillar spaces of the collagen matrix^32,38,39^. Furthermore, the NaOCl affects not only the organic phase but also the inorganic parts of the root canal dentin^40^. The erosive effect of NaOCl was more evident in the non-instrumentation heated NaOCl group indicating that the organic matter dissolving ability of the NaOCl increases with the temperature and confirms the results of previous studies^41,42^.

Changes in the chemical structure of dentin can affect its flexural and elastic strength that may jeopardize the success of the root canal treatment. Previously numerous studies evaluated the effect of different root canal preparation procedures on the microstructure of dentin and reported varied findings^17,30–32^. Previously, Moura-Netto et al.^43^ evaluated the effect of Nd:YAG laser irridation on the root canal dentin and reported that an observation of irregular and non-uniform surface with dentin fusion and re-solidification without smear layer and debris formation. In contrast, it was reported^44^ that 940 nm diode laser irradiation does not cause significant additional loss of mineral content of root canal dentin. Inconsistency between the studies could be explained by the fact that different type of lasers may affect the root canal dentin in different ways. Although it is possible for the laser irradiation to affect the mechanical and chemical properties of both the root canal sealers and dentine^45,46^, there is no information in the literature about the effect of direct blue light application on the chemical properties of root canal sealers and dentin. According to our findings, using blue light for root canal disinfection does not have any detrimental effect on the chemical component of root canal dentin. In contrast, NaOCl irrigation resulted in a decrease in the carbonate/phosphate and amide I/phosphate ratios which is in accordance with the findings of the previous studies^47,48^. This finding indicates that NaOCl not only deproteinizes the collagen on the dentin but also removes some carbonate ions from the inorganic dentin structure^49^ and confirms the claim that carbonate groups are more soluble than phosphate groups^50,51^. However, it didn’t change the Ca/P ratio significantly except for the heated NaOCl irrigation. In general, while the detrimental effect of NaOCl on the mechanical properties of dentin was explained by its organic component dissolving ability, the inorganic component of dentin was accepted to remain intact following NaOCl irrigation^52^. On the other hand, different studies have demonstrated that the inorganic content of dentin might also decrease after NaOCl irrigation^48,53^. Previous, studies^53^ evaluated the effect of different irrigating solutions on the mineral content change of root canal dentin and reported that NaOCl irrigation decreased the Ca/P ratio significantly. The inconsistency of the result of the present study with the previous ones can be explained by the methodological differences as the previous study^53^ performed 1-h NaOCl application on dentin. In contrast, it has been reported that 15 min irrigation with 5.25% NaOCl did not decrease the Ca/P ratio significantly^54^. The extended irrigation results in severe erosion of the dentin^55^. Heating NaOCl also increases its organic matter dissolving ability^41,42^.

The non-instrumentation + NaOCl + EDTA and laser groups were the only groups that had a similar chemical composition to the control group. In the non-instrumentation groups, the final flush of the root canals using EDTA recovered the carbonate/phosphate and amide I/phosphate ratios. However, the ratios decreased significantly following instrumentation and irrigation with NaOCl + EDTA, indicating that instrumentation renders root canal dentin more susceptible to the detrimental effects of irrigation solutions. Additionally, instrumentation causes a smear layer formation and the EDTA solution may have only reacted with the smear layer on the instrumented root canal surfaces and not able to recover the ratios as the reaction depth of the EDTA is time-dependent^56^. This may have resulted in more phosphate ions remaining on the root canal surface and decreased carbonate/phosphate and amide I/phosphate ratios in the Instrumentation + NaOCl + EDTA group. In the teeth apatite, carbonate groups may occupy hydroxyl and phosphate ions sites and the removal of carbonate from the surface would be easier than the subsurface groups, because the accessibility to the groups that are in deeper layers of dentin makes them less susceptible to the action of the solutions^40^.

The AH Plus is an epoxy resin-based sealer that penetrates into the dentinal tubules by sealer tags which considered as an important factor for mechanical bond. When the amino groups of dentinal collagen bond to the epoxy rings of the sealer, the chemical bond occurs^57^. Thus, it has been reported that the collagen-dissolving ability of NaOCl causes poor chemical bond between the sealer and dentin^57^. However, according to the SEM images, the AH Plus exhibited adequate marginal adaptation with the dentin irrespective of the preparation protocol without the formation of sealer tags. The sealer tag formation may not be an important factor for the adhesion of the sealer as previously it has been reported that the bond strength was not higher for the sealers that were able to penetrate inside the tubules^58^.

The BioRoot RCS is a tricalcium silicate-based that can form mineral infiltration zone by the formation of tag-like structures, intrafibrillar apatite deposition and apatite layers^59,60^. It has innovative properties like apatite-forming and calcium releasing ability^61^ which was confirmed by the findings of the present study that demonstrated higher Ca/P ratio in the BioRoot RCS group when compared with the AH Plus.

According to the finding of the present study, light application did not change either the carbonate/phosphate or the amide I/phosphate ratios both in the BioRoot RCS and AH Plus groups which confirms that using light for root canal disinfection does not have any detrimental effect on the chemical properties of dentin. However, heated NaOCl irrigation with instrumentation resulted in a considerably decrease in the carbonate/phosphate and the amide I/phosphate ratios both in the BioRoot RCS and AH Plus groups. This finding is in accordance with the report that NaOCl has an increased organic matter dissolving ability at higher temperatures^41,42^.

Blue light has a proven antimicrobial activity through the activation of endogenous photosensitizers which results in the generation of reactive oxygen species that kills bacteria by attacking their bacterial cell components^8,23^. Researchers^23^ applied 405 nm blue light on dentinal slices from a distance of 7 mm with different irradiance readings (470, 968, 1473, 1923, 2360, 2774, 3152, 3503, 3768, and 4054 mw/cm^2^) and reported that the light can be transmitted through the dentin. Then, they assessed the antibacterial efficacy of the light by irradiating agar plates including *S. mutans* for 15 min or 33 min using same settings and demonstrated that the blue light irradiation at doses of 340 and 831 J/cm^2^ led to significant reductions in bacterial growth and viability. Other research^8^ developed an implantable wireless blue micro light-emitting diode device and placed it into an infected root canal for 3 and 7 days. They reported that planktonic bacteria inside the root canal were eliminated by blue light irradiation with 432, 36 and 1.35 J/cm^2^. The different studies report different parameters thus no direct comparison can be made. An adjustment in the reporting of data to enable the working out of radiant exposure needs to be made.

In accordance with this report, the present study demonstrated that the application of light for at least 10 min with the 200 µm fibre and 5 min with 105 µm fibre resulted in an inhibition zone on the surface of agar plates. However, our findings revealed that light application could not obtain complete elimination of bacteria inside the root canal as there was no statistically significant difference between the light and negative control groups neither in the *E. faecalis* nor the *S. mutans* inoculated samples. It is well known that the antimicrobial action of blue light is dose-dependent and increases with the exposure time. The lower amount of dose and the shorter irradiation time of the light applied in the present study may explain the inconsistency of the findings between the present study and the previous research^8^. Researchers^8^ have applied the light into the root canals for 3 and 7 days, in the present study, the application time was shorter (15 min). However, increasing the power and exposure time results in a temperature rise above the biological limits as it is believed that a 10 °C increase is a critical level and a heat increase above this level may result in irreversible periodontal injuries^28,29^. To able to keep the temperature within the biological limits while obtaining an antibacterial affect, the light needs to be applied with lower power settings and longer application time or used a pulsed light so the temperature can reduce when the light is off. A longer treatment duration may not be acceptable from a clinical perspective because root canal treatment time is already last more than 1 h (74 min) for a molar tooth^62^.

According to the result of the present study, NaOCl irrigation significantly reduced the number of CFUs inside the root canal both in the instrumentation and non-instrumentation groups. This finding has proved the strong antibacterial efficacy of NaOCl which is in accordance with previous results^5,63^. Interestingly, the instrumentation and distilled water irrigation protocol also removed bacteria from the root canal space effectively which demonstrates that instrumentation is also an important factor in the removal of bacteria from the root canal system and can provide similar results to the NaOCl irrigation. In accordance with this result, Byström et al.^64^. reported that mechanical instrumentation with saline irrigation can reduce the number of bacteria considerably. Thus, it can be asserted that instrumentation causes mechanical disruption of biofilms inside the root canal systems and accompanying distilled water irrigation flushes away the disrupted biofilms from the root canal space.

One of the limitations of the present study would be that extracted human teeth with different root canal morphology, size and anatomic variations were included. To overcome these limitations, teeth with similar root sizes were distributed into the groups equally. *In-vitro* studies cannot mimic the exact clinical situation but they can assist in revealing the effect of root canal preparation on dentin^13^.

It is known that light irradiation causes a significant reduction in the proliferative activity of gingival fibroblasts, increases in intracellular ROS levels. Additionally, intracellular mitochondrial disorders can be observed following blue light application. However, in the present study, the light was applied into the root canal in which any oral tissues or cells were not present. Nonetheless, the clinicians should be aware of the detrimental effect of light application on the oral tissues and cells. In our study, the irradiance was 38,072 and 1032 mW/cm^2^ for 105 and 200 µm fibres, respectively.

## Conclusions

Instrumentation and NaOCl irrigation have detrimental effects on the chemical structure of dentin, and it was more evident when NaOCl was heated. Furthermore, intracanal heating results in a rise in the temperature that is higher than 10 °C on the root surface. Minimally invasive root canal preparation techniques where the root canal is not instrumented and is disinfected by light irradiation followed by obturation with a hydraulic cement sealer may be an attractive treatment option for management of vital teeth needing root canal therapy. The blue light could be transmitted to a depth of 4 mm, does not result in a temperature rise higher than the biological limits when used in the presence of distilled water in the root canal and does not have any detrimental effects on the chemical structure of dentin. Blue light is a promising tool due to its easy use and antibacterial features. Using blue light for root canal preparation should be further evaluated in prospective clinical studies.

## Materials and methods

### Tooth preparation

**Figure 8:**
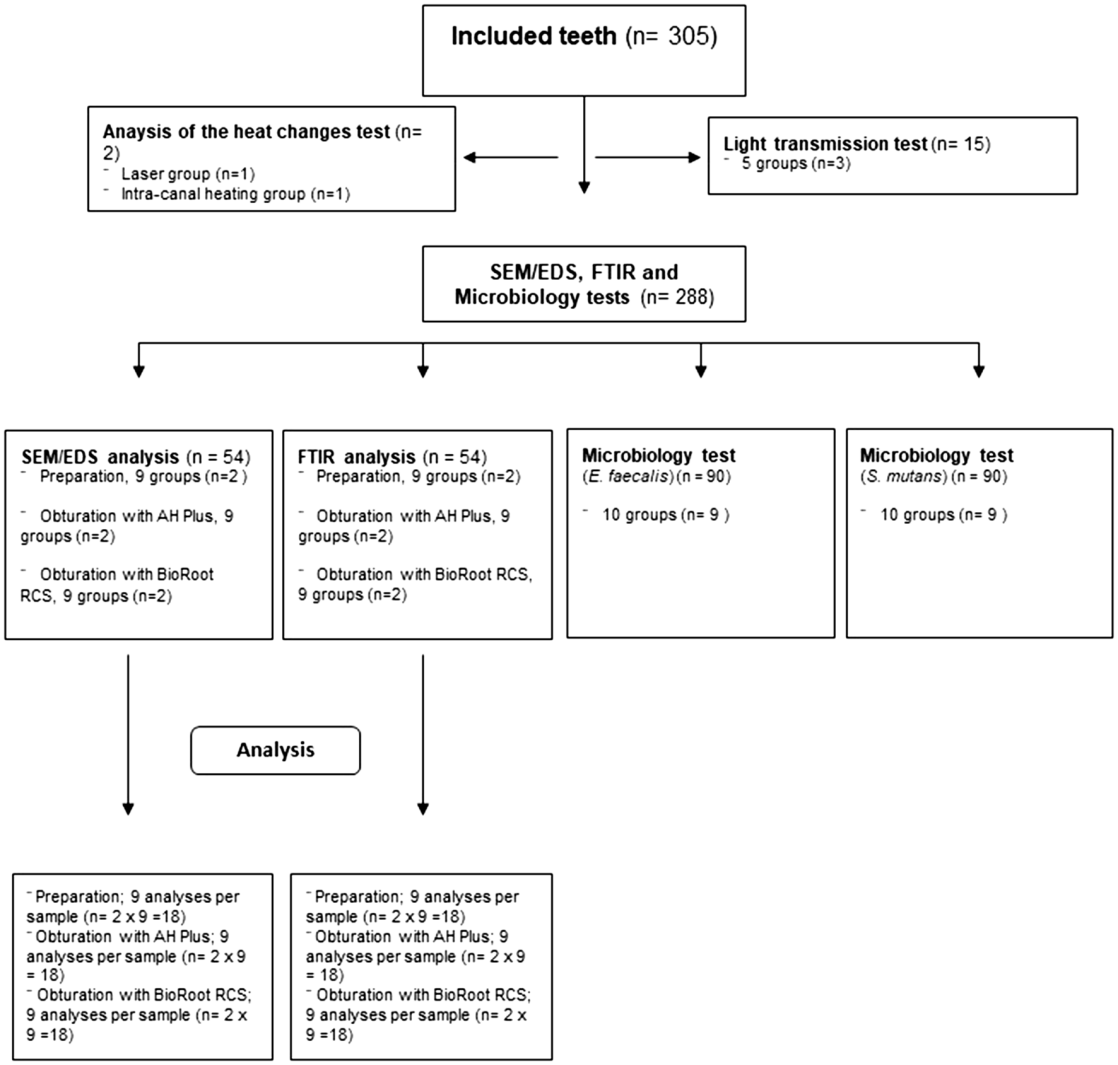
Distribution of teeth according to the groups.

A power analysis was conducted using GPower program (Franz Faul, University of Kiel, Germany) based on the data obtained from a previous study^65^. The analysis revealed that 5 analyses were sufficient per group (α = 0.05, power = 0.8, effect size = 0.643). Three hundred and five extracted human single rooted teeth (maxillary central incisors, lateral incisors, maxillary and mandibular canines and premolars) were obtained from the University of Birmingham Dental School Tissue Bank. Ethical approval was granted from the Research and Innovation Department, Birmingham Community Healthcare Trust (14/EM/1128). The teeth involved in the research were extracted for reasons other than the current research. The teeth were randomly distributed in all experiments. After the soft tissue and calculus remnants on the surface of the roots were removed mechanically using a scaler, the teeth were decoronated with a diamond disk to obtain a standardized root length of 15 mm^66^ since the average root length of single rooted teeth was reported within a range of 12.2–16.6 mm^67^. Two hundred and eighty-eight specimens were then embedded in dental impression putty. Figure 8 shows the distribution of the teeth according to the groups. After the setting of the resin, the teeth were removed from the putty and longitudinally sectioned into two halves using Isomet low-speed saw (Isomet, Buhler, Lake Buff, USA). Then, the two halves of the sectioned teeth were realigned and wrapped by using Parafilm and finally placed into the previously numbered putty. The samples were divided into 9 groups (n = 32). The teeth with similar root sizes were distributed into the groups equally. The samples were preserved in a vacuum during the study.

Root canal therapy was undertaken using the standard root canal preparation and irrigation as follows using Reciproc files (R25 and R50) with an electric motor (AI Endomotor, Guilin Woodpecker Medical Instrument Co Ltd) at a working length of 14 mm. The irrigation protocol was varied in the different 9 groups as follows:

- *Distilled water:* The root canals were irrigated with 10 mL of distilled water by using a side-vented irrigation needle.
- *NaOCl group:* The root canals were irrigated with 1 mL of 5.25% NaOCl after every three pecking motions by using a side-vented irrigation needle. A total of 5 mL 5.25% NaOCl was used during the preparation. Then, the final irrigation was performed with 5 mL of 5.25% NaOCl for 3 min. A total of 10 mL 5.25% NaOCl was used.
- *Intra-canal heated NaOCl group:* After the instrumentation of the root canals, final irrigation was performed by using 5 mL of NaOCl solution. Intra-canal heating was performed as described previously.
- *NaOCl* + *EDTA group:* all the procedures were the same as in group 2 except the final irrigation with 5 mL of 5.25% NaOCl was followed by 5 mL of 17%EDTA irrigation for 3 min.

Another 4 groups were formed as the non-instrumentation groups. The non-instrumentation groups excluded the use of the reciprocating files and used the same irrigation protocol as the instrumentation groups and a laser group. Finally, ninth group was formed as the laser group. For the laser group, the light was applied from both the inside the root canal and outside the root canal. Before the application of the light, the root canal was filled with 0.1 mL of distilled water. Then, a 200 µm fibre (FP200ERT, Thorlabs Inc., New Jersey, USA) was inserted into the root canal to 5 mm from the apex and the laser device was activated at the highest power setting of 1032 mW/cm^2^. Since the light was applied for 10 min, the total radiant exposure was 619 J/cm^2^. Details about the light application (light source, powers settings and irradiance) mentioned below in preliminary testing of the light group section.

For the application of the light outside the root canal, a 105 µm fibre was used. The light was applied from the access of the root canal for 5 min with the highest power setting of 38,072 mW/cm^2^ for this fibre. The total radiant exposure for this fibre was 11,421 J/cm^2^. Finally, the root canals were irrigated with 10 mL of distilled water by using a side-vented irrigation needle.

### Root canal obturation

Seventy-two extracted human teeth with a single root canal were selected and standardized to a root length of 15 mm. Then, all the teeth were divided into 9 different root canal preparation techniques described previously (n = 8). After the completion of the root canal preparations, all the samples were subdivided into 2 groups obturation groups (n = 4); AH Plus (Dentsply DeTrey, Konstanz, Germany) (n = 2) or BioRoot RCS (Septodont, Saint-Maur-des-Fosses, France) (n = 2). The sealers were prepared according to the manufacturer’s recommendations, and they were dispensed in the root canal using gutta-percha points. A cold lateral compaction technique was used for filling all the root canals and the samples were stored in an incubator at 37 °C for 7 days. Then, the roots were sectioned horizontally using Isomet low-speed saw, and 3 dentin slices (coronal, middle, and apical) were obtained for each sample.

### Preliminary testing of the light transmission group

#### Light transmission

**Figure 9:**
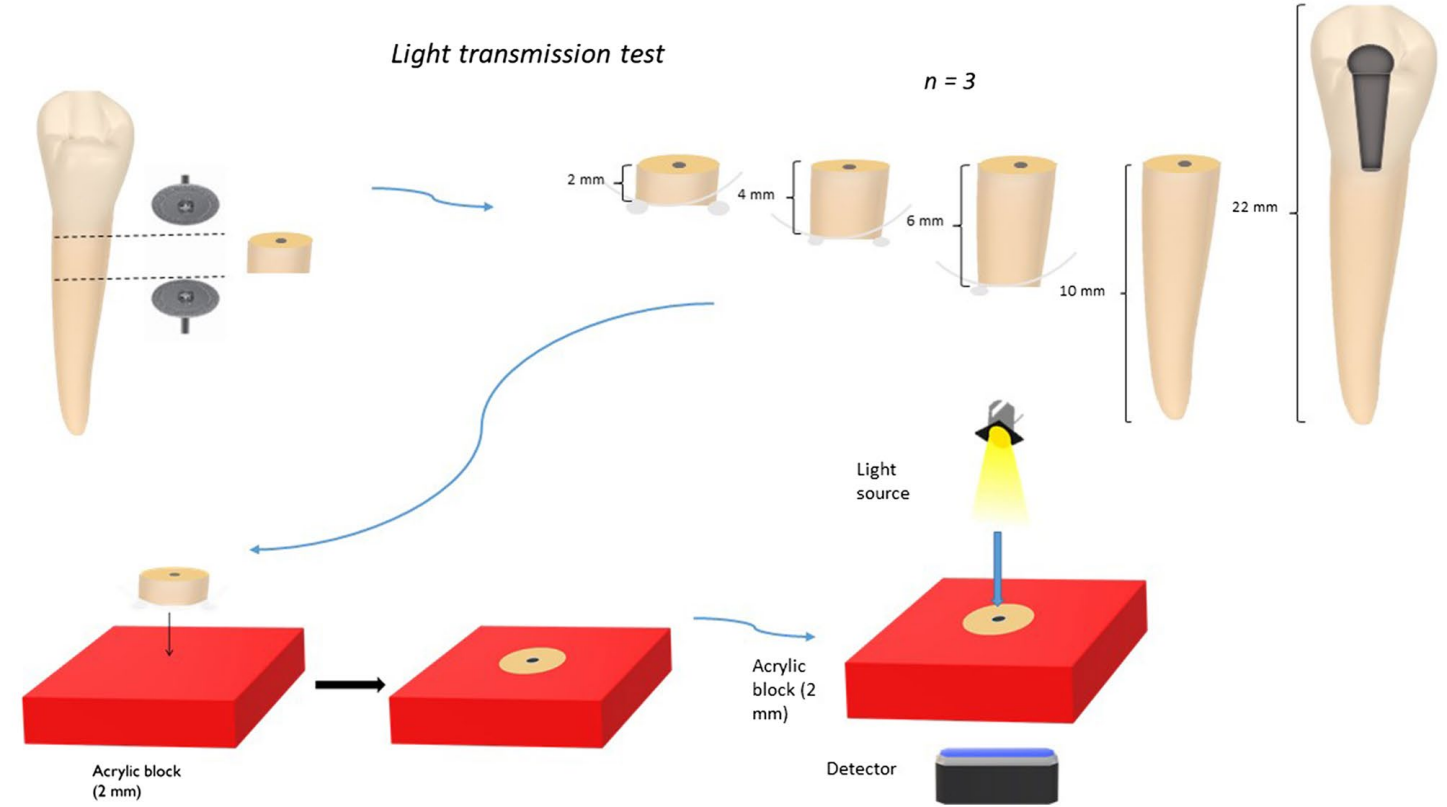
A schematic presentation of the light transmission test.

A total of 15 extracted human teeth with a single root canal were randomly selected. After the soft tissue and calculus, remnants on the surface of the roots were removed mechanically using a scaler, all the teeth were embedded vertically into 10 × 10 mm acrylic blocks. Then all the samples were randomly distributed into 5 different groups (n = 3) and sectioned horizontally with a diamond disk to obtain root lengths of 2 mm (group 1), 4 mm (group 2), 6 mm (group 3), 10 mm (group 4), and 22 mm (group 5)^23^. All surfaces of the acrylic blocks, except the coronal and apical surfaces of the roots, were painted with black varnish to allow light to transmit through the dentin, not the acrylic (Fig. 9). After calibration of the spectrometer (USB4000-VISNIR, Ocean Optics, FL, USA) connected with 400 µm optical fibre (Ocean Optics Inc.) and a glass cosine corrector (3.9 mm; CC3, Ocean Optics, FL, USA) using deuterium/halogen light source DH2000 (Ocean Optics Inc.), the transmission of light was measured with the acrylic block aligned between the detector below, and the light source above^23^. Decreasing irradiation power settings were applied reducing in 10 percent increments from 100 to 10%. A further 2 additional measurements were taken at 5% and 2%. A total of 12 irradiance readings (mW/cm^2^) were obtained, each measurement was recorded in triplicate, and an average value was calculated. This was done to generate a calibration curve at each thickness to accurately determine the irradiance delivered.

#### Laser fibre irradiance measurement

A 105 µm (Ocean Optics Inc.) and a 200 µm fibres and a laser (MDL-III-405-FC-250mW, Changchun New Industries, China) with a wavelength of 405 nm were used. The irradiance levels were calculated for both 105 and 200 µm fibres after measuring the power using a power meter (PD300R, Ophir Optronics Solutions Ltd., Jerusalem, Israel) and StarLab 3.0 software (Ophir Optronics Solutions Ltd.). The calculation was done by using fiber diameters. Three readings for each fibre were obtained and an average value was calculated.

#### Analysis of the heat changes

The heat generated by the light application and also by the heat carrier in the heated sodium hypochlorite group was investigated by assessing the heat changes inside the root canal and also on the external surface of the tooth.

*Light group;* three extracted human teeth with a single root canal were selected and prepared as described previously. A gelatinized HBSS colloidal gel was prepared by mixing HBSS with 20% porcine gelatin (Biochemika Fluka, Sigma-Aldrich) and the teeth were embedded into the colloidal gel up to their coronal level.

The temperature of both the external root surface and inside the root canal were measured. To measure the intra-canal temperature changes, side canals were drilled 5 mm from the apex of the teeth, and the K-type thermocouple (1.5 mm tip diameter) (PICO Technology, Cambridgeshire, United Kingdom) was then inserted into the artificial side canals. For the external root surface measurements, 3 K-type thermocouples were attached to the coronal (10 mm from the apex), middle (6 mm from the apex), and apical (2 mm from the apex) levels of the root using a tape to ensure good contact between the tip of the thermocouples and the root surface^68^. Another fourth thermocouple was placed in the colloidal gel to record the temperature of the gel. Then, the 200 µm fibre was inserted into the empty root canal at a level of 5 mm from the apex. The laser device was activated for 30 s and deactivated for 30 s (5 times each), and the heat changes were recorded over a 10 min cycle. The experiment was repeated using the same teeth and the same experimental setup but with the presence of 0.1 mL of distilled water inside the root canal. Finally, temperature changes were calculated for both experiments.

*Intra-canal heated NaOCl group;* A group was included using intra-canal heat application during the irrigation of the root canals. Heat changes were measured inside and outside the root canal system during the use of heated irrigation to determine safety.

The irrigation was performed with a total of 10 mL of 5.25% NaOCl. First, the irrigation of the root canals was performed by using 5 mL of NaOCl. Then, another 5 mL of NaOCl solution was used for the intra-canal heating procedure. The intra-canal heating was done for 10 secs with XF-tip placed 3 mm short of the working length by using a heat source (SuperEndo α, B&L Biotech, Philadelphia, United States) that was set at 200 °C. The intra-canal heating regimen was repeated 5 times (10 s each) with 1 mL of 5.25% NaOCl replenished for every cycle. A total of 10 mL of NaOCl solution was used.

The same experimental setup was used as previously described. Following irrigation of the root canal using 1 mL of 5.25% NaOCl, intra-canal heating was performed for 10 s with XF-tip placed 3 mm short of the working length by using a heat source (SuperEndo α, B&L Biotech, Philadelphia, United States) set at 200 °C. This procedure was repeated 5 times and temperature changes were recorded. Additionally, this experiment was repeated with distilled water irrigation instead of the NaOCl, and without any irrigation (dry canals).

### Characterization of root dentin after root canal preparation

Thirty-six extracted human teeth with a single root canal were included. The roots were divided into 2 groups for either scanning electron microscope (SEM)/energy dispersive spectroscopy (EDS) analysis or Fourier-transform infrared (FT-IR) spectroscopy analysis and then subdivided randomly into 9 groups according to chemo-mechanical preparation procedures described previously (n = 2 for SEM/EDS, n = 2 for FTIR).

#### SEM/EDS analysis

Both prepared and obturated specimen halves were sputter‐coated with gold, and examined under the SEM (SEM; Zeiss MERLIN Field Emission SEM, Carl Zeiss NTS GmbH, Oberkochen, Germany)^65^. First, the full view of each specimen was scanned at low magnification (×60). Then the secondary electron images of coronal, middle, and apical were taken at ×3000 magnification at a working distance of 8.5 mm.

For EDS analysis, the following parameters were used, EHT = 20 kV, Iprobe = 1000 pA and WD = 8.5 mm for a 35° take off (elevation angle). EDS analyses were performed at 3 different areas at the coronal, middle, and apical levels. Thus, a total of 9 analyses were obtained for each sample (n = 18). The alterations of the dentin surface by different preparation procedures were measured by monitoring changes in the calcium/phosphorus (Ca/P) ratio^65^.

#### FT-IR microscopic analysis

FT-IR microscope was used to determine compositional changes on human dentin after each irrigation protocol^65^. The analyses were performed at 3 different areas at the coronal, middle, and apical levels. Thus, a total of 9 analyses were obtained for each sample using a Nicolet iN10 FT-IR machine (Thermo Scientific Instruments Corp., Madison, WI, USA) and Omnic 8 software suite (Thermo Scientific Instruments Corp.) at a resolution of 8 and 16 scans (n = 18). After scanning, the baseline tracing was performed, and the areas under the infrared band amide I (1600–1700 cm^−1^), phosphate (1170–780 cm^−1^), and carbonate (888–816 cm^−1^) were calculated by Microsoft Excel. Subsequently, the ratio of the amide I/phosphate was determined indicating the organic components of dentin. The inorganic components were calculated by the carbonate/phosphate band area ratios. Carbonate/phosphate and amide I/phosphate ratios were calculated for all the groups and the ratios were compared among the groups.

### Microbiological tests

#### Agar diffusion assay

Agar diffusion assays^69^ were performed to demonstrate the antibacterial effect of light. *Enterococcus faecalis* (*E. faecalis*) ATCC 29,212 was retrieved from frozen stocks (Oral Microbiology Group, School of Dentistry, UoB) and incubated for 24 h on brain heart infusion (BHI) agar (SigmaAldrich®, USA) at 37C. Then, 3 random morphologically similar colonies were inoculated into 5 mL of BHI broth (SigmaAldrich®, USA) and incubated at 37 °C, %100 air atmosphere overnight in a shaking incubator at 100 rpm (NB-205, NBiotek, Korea). After the incubation period, a bacterial lawn was created on the BHI agar surface using sterile cotton swabs. Then, the light was applied to the surface of the agar plate with a Laser (MDL-III) using either a 105 µm or 200 µm fibre. Since root canals with a height of 15 mm (14 mm working length) were planned to be used in the study, it was decided to apply light with a 105 µm fibre up to 10 mm into the coronal part of the canal and to use a 200 µm fibre to reach 4 mm into the apical part of the root canal. Because the 105 µm fibre had a metal jacket with outer diameter larger than the diameter of the root canal and therefore it was physically impossible to reach the 144 mm depth. The 200 µm fibre had no jacket and the outer diameter of the fiber was smaller than the diameter of the root canal. Thus, this experimental setup utilized light applied to the agar surface at a distance of 4 mm using the 200 µm fibre and from a distance of 10 mm using the 105 µm fibre. To determine the minimum required irradiance to inhibit bacteria, the light was applied for 2, 3, 5, and 10 min. All the experimental procedures were performed in a flow hood. After light exposure, the agar plates were incubated for 24 h at 37 C. The resulting diameters of the zones of inhibitions were measured under an optical microscope (Primotech, Zeiss, Germany) at a magnification of ×2.5.

#### Optical density and colony forming units (CFUs) correlation

*E. faecalis* ATCC 29212 and *Streptococcus mutans* (*S. mutans)* 3209 were used for microbiological experiments^70,71^. Overnight cultures were prepared by inoculating 3 colonies of either *E. faecalis* or *S. mutans* into 5 mL of BHI broth each and incubated in the shaking incubator as described earlier. After the incubation, the optical density at 600 nm (OD_600_) was measured. Then, the overnight cultures were diluted using BHI broth to obtain 4 different suspensions for each species (*E. faecalis* and *S. mutans*) with an OD_600_ of 1.063, 0.744, 0.496, and 0.265 for *E. faecalis,* and an OD_600_ of 1.039, 0.528, 0.302, and 0.174 for *S. mutans.* These dilutions were then used to plate CFUs. This allowed a correlation between optical density and CFUs.

Two hundred microliters (µL) of the solutions were added into wells of a 96-well plate and tenfold serial dilutions in phosphate-buffered saline (PBS) were performed, Then, 20 µL aliquots of the cultures were plated on BHI agar plates, they were incubated at 37 °C for 24 h and the CFUs were counted. For the inoculation of the root canals, 10^8^ CFUs/mL bacteria were used which equals an OD_600_ of 0.23 for *E. faecalis* and 0.06 for *S. mutans*.

### Assessing the antibacterial effect of different root canal preparation procedures

One hundred and eighty extracted human teeth with a single root canal were included in the study. Teeth that had more than a single root and/or canal, previous root canal treatment, external/internal root resorption, immature root apices fractures/caries/cracks on the root surface, and/or root canal curvature more than 5° were excluded. After the soft tissue and calculus remnants on the surface of the roots were removed mechanically using a scaler, the teeth were decoronated with a diamond disk to obtain a standardized root length of 15 mm. The specimens were then embedded in dental impression putty in 1.5 mL Eppendorf tubes sterilized by autoclave at 121 °C for 20 min. The teeth were divided randomly into 2 groups for infection with either *E. faecalis* or *S. mutans*. Then the specimens were subdivided into 10 groups according to the types of chemo-mechanical preparation that were to be investigated.

Bacterial suspensions of 10^8^ CFUs/mL were prepared using a correlation of OD_600_ and CFUs and used for the inoculation of the root canals^72^. The root canals were filled with 20 µL of the bacterial suspension by using a pipette and a 27-gauge side vented irrigation needle inserted into the root canal 1 mm from the apex (14 mm). Then the samples including *E. faecalis* were incubated for 24 h and those containing *S. mutans* for 48 h at 37 °C as. Both instrumentation and non-instrumentation groups were assessed. For the negative control neither instrumentation nor irrigation was performed. After the completion of the root canal preparations, the roots were removed from the dental impression putty and placed into Eppendorf tubes including 1 mL of PBS. Then the tubes containing the samples were sonicated for 1 min in an ultrasonic device (Vitasonic, Vita Zahnfabrik, Bad Säckingen, Germany). Two hundred microliters (µL) of the solutions were added into the wells of a 96-well plate and after twofold serial dilutions, the suspensions were plated and incubated as described previously. Then the CFUs were counted.

### Statistical analysis

The Kolmogorov–Smirnov test was used to analyse the normality of the all data. While the Kruskal–Wallis and the Mann–Whitney U tests were used for the analyse of the data which was not normally distributed, the One-Way ANOVA and Tukey post-hoc tests were used for the analyse of normally distributed data. All the analyses were performed at a 95% confidence level.

## Data Availability

The authors confirm that the data supporting the findings of this study are available within the article.
